# Why downsizing may increase sickness absence: longitudinal fixed effects analyses of the importance of the work environment

**DOI:** 10.1186/s12913-025-12454-w

**Published:** 2025-02-28

**Authors:** Anniken Grønstad, Vilde Hoff Bernstrøm

**Affiliations:** 1 Oslo New University College, Ullevålsveien 76, Oslo, N-0454 Norway; 2 Work Research Institute, OsloMet, Oslo Metropolitan University, Stensberggata 26, Oslo, N-0170 Norway

**Keywords:** Downsizing, Absenteeism, Sickness absence, Organizational change, Work environment, Mediation

## Abstract

**Background:**

Downsizing can often have a detrimental effect on employee health and increase sickness absence. Earlier research has theoretically argued that such negative consequences are due to taxing alterations in the work environment, but research efforts to empirically test this argument remain limited.

**Methods:**

In this study, we investigate whether the environment for control, role clarity, and commitment in different work units can explain the relationship between unit-level downsizing and sickness absence. We combined register- and self-reported data from 19,173 employees in a large Norwegian health trust in the period 2011–2015 and conducted a longitudinal fixed effects analysis.

**Results:**

Unit-level downsizing was found to be significantly related to increased short-term sickness absence, reduced organizational commitment, and reduced control. Reduced commitment explained a small part of the increase in short-term sickness absence after unit-level downsizing. There was no mediating effect of either control or role clarity.

**Conclusion:**

The study contributes to a better understanding of the underlying mechanisms that help explain why downsizing leads to adverse health consequences and sickness absence by highlighting the complexity of this relationship and introducing organizational commitment as a relevant mediator.

**Supplementary Information:**

The online version contains supplementary material available at 10.1186/s12913-025-12454-w.

## Background

Downsizing is increasingly implemented in Scandinavian health trusts as a response to the demands of shrinking budgets and financial expectations and performance pressure [1, 2]. However, a salient downside of downsizing is its adverse influence on important employee outcomes, such as their health and well-being [3]. Studies show that healthcare employees experiencing downsizing have higher rates of sickness absence [4, 5] and that downsizing also diminishes the likelihood of work resumption among already absent employees [5]. In addition, downsizing has been related to reduced worker well-being [6], deteriorated physical or mental health [7–10], higher odds of medication use [11], higher prevalence of disability pension rates [12] and upticks in cardiovascular disease, drug prescriptions and hospital admissions [13]. By contrast, some studies have found no significant effect of downsizing on adverse employee health outcomes [14, 15].

To promote workplace health and reduce the adverse health effects of downsizing, it is important to understand why downsizing may lead to adverse outcomes. Indeed, identifying underlying mechanisms can help to improve and govern the downsizing process and help human resources departments (HR) to retain and reinforce critical change components that influence the workforce.

A theoretical argument has been proposed that change in itself may not be hazardous, but that specific organizational changes, such as downsizing, will often affect employees’ health through alterations in their work environment [16–18]. Arguably, the content and process of downsizing will often bring about changes in the work environment, and if these changes are experienced negatively, they are likely to have adverse effects on employee health and sickness absence.

While several authors have argued that organizational change is associated with adverse health and absence effects due to changes in the work environment, few studies have statistically tested the association, and only for a limited number of work environment factors [19]. So far, studies have supported the mediating role of job insecurity, high demands, low job control, social support and procedural justice in the relationship between downsizing and employee health-related outcomes [3, 18, 20, 21]. Similarly, investigating related organizational changes such as restructuring and employee health outcomes, studies have also supported mediators such as job insecurity and work intensity [6, 22]. The importance of these variables as mediators between organizational change and health outcomes varies according to the different forms of organizational change [21], and the type of health outcomes [22]. The degree to which the variables collectively explain the relationship between downsizing and adverse outcomes varies, with estimates ranging from 10 to 49% [18, 21].

**Fig. 1:**
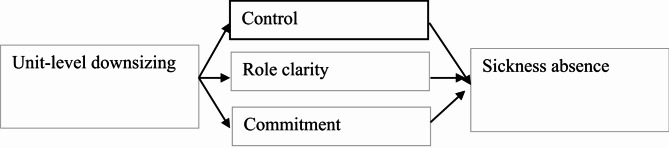
Theoretical model

Furthermore, several qualitative studies support the importance of multiple work environment factors as mediators between downsizing and other types of organizational change and health and absence. For example, research has investigated changes including downsizing [23] as well as transformational change such as change in individual roles [17] describing how these changes could lead to disrupted and insecure social bonds, increased demands and incompatible expectations, lack of confidence in the employer and in colleagues, and eventually burnout and sickness absence. Collectively, there is support for the work environment as a mediator between organizational change such as downsizing and health and absence outcomes, but there is a clear need to broaden the scope of work environment factors included in this relationship. To contribute to a broader understanding of why downsizing leads to adverse employee health outcomes, the present study empirically tests three potential work environment mediators in the relationship between unit-level downsizing and sickness absence: control, role clarity and organizational commitment, as presented in Fig. 1. Role clarity and organizational commitment have not previously been empirically investigated as mediators in the relationship between downsizing and employee health and absence outcomes. However, as we argue below, both are theoretically expected to be important health-damaging consequences of downsizing. A perceived sense of control has been investigated as a mediator in previous studies, but with substantial variations in the degree of explained variance [18, 21].

### Unit-level downsizing in a Norwegian health trust

The present study focuses on unit-level downsizing within the health care sector, defined as a reduction in personnel in a work unit of at least 20% during one quarter. The health and social sector is one of the largest employers in Norway, accounting for around 20% of the country’s workforce [24]. Unit-level downsizing is a relatively frequent form of organizational change within the sector [5], making it an imporant change to understand.

Within the healthcare sector, changes, such as downsizing, may take a range of structural forms at various organizational levels including organizational, departmental, or unit-level. In the Norwegian healthcare sector, the pooling of hospitals under state ownership [25] has propelled the rate of change at the organizational level, and these changes have generated a higher pace of smaller and internal changes, such as unit-level downsizing [26–28]. Noteworthy, downsizing often occurs without layoffs [5]. Instead, the health trust employs strategies like not renewing temporary contracts, not filling vacancies, and transferring workers.

The downsizing investigated in this article takes place at the unit-level, which is defined as the lowest organizational level in the health trust. The organizational architecture of a health trust consists of the overall organization which in turn is made up of a hierarchy of several smaller units within the health trust (e.g. devisions, departments and teams). We focus on units at the lowest organizational level in the health trust. While the health trust investigated in the current study employs several thousand employees, each unit investigated employs on average approximately 20 workers, resembeling a small (and in some instances midsize) company in size.

In large organizations there will be instances where some units remain stable (i.e., no change) whereas others expand, and yet other units downsize without having the same change simultaneously taking place at higher organizational levels. During the study period the number of employees within the health trust being investigated remained stable. However, different units within the health trust upsized, downsized, or remained stable. Indeed, despite no general decline in the number of employees at the health-trust, unit-level downsizing was a frequent type of change [4].

Previous research on downsizing has, to a wide extent, concentrated on the organizational level [29] and the consequences of organization-wide changes [19, 26, 30–33]. However, more recent studies have reported negative health consequences and sickness absence resulting from changes at the work-unit level [16, 21, 28, 34, 35]. Specifically, studies have documented increased sickness absence following unit-level downsizing [4] and lay-offs [16]. In contrast, Fløvik et al. [21] did not observe a significantly increased risk of mental distress related to downsizing or layoffs at the unit–level. The negative consequences of such frequent changes at the unit-level warrant a specific focus on understanding the mechanisms explaining them.

## Theory and hypotheses

### Perceived control in the work units

The first potential mediator is the level of employee control in the work unit. Employee control, which is also referred to as decision latitude, contains two facets: decision authority– which is conceptualized as an employee’s ability to make decisions in their work - and skill discretion– which reflects the degree to which employees can use certain skills when performing their job [36]. Earlier research highlights control as an important work-related factor that influences employees’ sickness absence [37, 38]. Control is a key component in the influential Job Demands-Control model [36, 39], which postulates that employees with high control over how to perform their work are less prone to job strain.

Multiple authors have argued that control is a potential mediator in the relationship between organizational change and adverse health effects and increased sickness absence [16, 18, 21]. There is also empirical support for control as a mediating variable between downsizing, and subsequent health and sickness absence outcomes. Kivimäki et al. [18] demonstrate that skill discretion, a central aspect of job control, mediated the relationship between major downsizing and long-term sickness absence. Job control accounted for 12% of the increased absence. More recently, Fløvik et al. [21] found that the relationship between work-unit level reorganization and mental distress was reduced when adjusting for the work factors job control, job demands and support. However, the explained variance was substantially lower than in the study by Kivimäki et al. [18].

If downsizing affects employees’ perceptions of control, control may in turn influence sickness absence in various ways, such as by impacting employee health, increasing employees’ possibility of continuing to work despite health impairments, and affecting employees’ motivation for attending work despite having reduced health. The importance of control for employees’ health and wellbeing is well documented [40–43]. At the same time, control could also provide the necessary flexibility for workers to remain occupationally active while experiencing health problems. Indeed, a large pool of research shows that work-related adaptations are important to support those suffering from health impairments to remain in some degree of employment [44] and that the employees themselves hold the key to identifying stressors and enablers in the workplace [45].

Based on these theoretical and empirical findings, we have formulated the following hypotheses:

- H1a: Unit-level downsizing is related to a reduced level of employee control in the work unit
- H1b: The level of employee control in the work unit is related to increased odds of sickness absence
- H1c: The level of employee control in the work unit will mediate the relationship between unit-level downsizing and short-term sickness absence

### Perceived role clarity in the work units

The second potential mediator is role clarity in the work unit. Role clarity concerns employees’ experience of clarity regarding their responsibilities and what is expected from them at work [46]. The concept of role clarity has also been considered as absence of role ambiguity [47]. Hence, role clarity can represent a resource in situations where employees are confronted with high demands (i.e., downsizing), since it produces clear expectations [48].

Greater role clarity is generally regarded as positive for health, thus reducing sickness absence. An absence of role clarity, on the other hand, can evoke uncertainty among employees as regards their responsibilities and what is expected of them at work, a situation that can be a source of considerable stress and is not uncommon in relation to organizational change [42]. Studies support the view that role clarity relates to short-term and long-term sickness absence [37, 49–51]. Additionally, role clarity is linked to health consequences such as psychological and physical strain [48], depression [52], burnout [53], and stress [54]. Robinson and Griffiths [17] identified uncertainty and ambiguity in roles as a central source of stress during organizational change, and most of the individuals they interviewed were waiting to find out how their role would change. In a similar vein, Verlinden et al. observed that role clarity declined in relation to change in the public sector as consequences such as uncertainty regarding work roles, responsibilities, and tasks surfaced, all of which may generate turbulence for individual workers, particularly at the work-unit level [55]. In this manner, unit-level downsizing might lead to increased sickness absence because it reduces the level of role clarity in the unit. Given the healthcare context of the present study, this is particularly relevant since the higher prevalence of complex change in public sector organizations (e.g., healthcare) and its ramifications for role clarity have received minimal scholarly attention so far [55].

To our knowledge, no studies have tested role clarity as a mediator between organizational change and adverse sickness absence consequences. However, Pollard [56] found that employees’ level of role ambiguity following organizational changes was significantly related to a greater decline in mental health and increase in systolic blood pressure.

Based on these theoretical and empirical findings, we have formulated the following hypotheses:

- H2a: Unit-level downsizing is related to the level of role clarity in the work unit
- H2b: The level of role clarity in the work unit is related to the odds of short-term sickness absence
- H2c: The level of role clarity in the work unit mediates the relationship between unit-level downsizing and short-term sickness absence

### Perceived commitment in the work units

The third potential mediator is the level of commitment to the organization identified in the work unit. The conceptualization of commitment applied in this study resembles affective commitment, which is described as an employee’s desire to stay in the organization owing to a harmonious relationship between the work expectations and experiences of the employees [57, 58]. Arguably, commitment can influence employees’ odds of sickness absence by affecting their health as well as their attendance motivation.

Commitment can protect employees from the adversity of demanding situations, such as a change event, since being highly committed reinforces perceptions of belongingness, stability and security, and diminishes job displeasure [59, 60]. Commitment to work may also motivate employees to attend work and refrain from being absent for minor health complaints [61]. Hence, a work environment characterized by high levels of commitment can both protect employees from strain and encourage attendance motivation.

Empirical studies support the view that higher commitment relates to reduced absence [62, 63]. In a meta-analysis, Meyer et al. [62] found that affective commitment was negatively related to absenteeism, as well as self-reported stress. Similarly, Hausknecht et al. [63] found that organizational commitment measured at the unit level was negatively associated with absence, showing that as commitment increased in the unit, absence decreased [63]. However, some studies have also yielded more mixed results. Clausen et al. [64] found support for a curvilinear relationship between commitment and absence among Danish eldercare workers. While medium levels of commitment had protective effects against long-term sickness absence, high levels of commitment did not. Schalk [65] found a significant relationship between commitment and health complaints, but no direct relationship between commitment and absenteeism.

Organizational change also has the potential to undermine employee commitment [66]. Downsizing in particular is a type of change that can influence thebond between the organization and the individual employee [66] and can be expected to lower the level of organizational commitment. Indeed, earlier research has shown lower levels of organizational commitment among downsizing survivors than among workers unaffected by downsizing [67].

During an organizational change such as unit-level downsizing, the employees’ commitment to the organization may be challenged by alterations of valued aspects of work and the organization. For example, when the change leads to work overload, role ambiguity, or conflict, employees are likely to become less committed [66]. The findings of Knudsen et al. [67] suggest that lower levels of organizational commitment among downsizing survivors were partially due to the perception of less organizational support, lower job autonomy and higher job pressure among survivors. Additionally, commitment may be further reduced when the change violates the reciprocal nature of an employer-employee relationship [66]. For example, downsizing could be conceptualized as a violation of the reciprocal relationship between employers and employees because employees are requested to maintain their commitment to and perhaps work even harder for an organization, which, by its very act of downsizing, has failed to demonstrate commitment to its employees [68]. Furthermore, as Meyer et al. [66] argue, employees could perceive the organization’s changes, which promote the organization’s interests but could prove to be detrimental to employees, as a violation of their trust. In this sense, unit-level downsizing could lead to increased sickness absence because it reduces the level of organizational commitment in the work unit.

To our knowledge, no studies have tested commitment as a mediator between organizational change and adverse health and absence consequences. However, previous research focusing on the mediating potential of commitment has found that it mediates the pathway from organizational stressors to employee health [69].

Based on these theoretical and empirical findings, we have formulated the following hypotheses:

- H3a: Unit-level downsizing is related to the level of organizational commitment in the work unit
- H3b: The level of organizational commitment in the work unit is related to the odds of short-term sickness absence
- H3c: The level of organizational commitment in the work unit mediates the relationship between unit-level downsizing and short-term sickness absence

## Methods

### Participants and sample

We analyzed data from a large Norwegian health trust in the third and fourth quarters of 2011, 2012, 2013, and 2015. Following a merger in 2009, the health trust had more than 20,000 employees dispersed between multiple locations in the capital of Norway. The health trust is highly specialized with local, regional and nationwide patient treatment responsibility.

The sample contained 27,806 unique employee numbers nested within 1,664 units. We restricted the sample to include full-time employees only ( > = 80%). We excluded work units consisting of less than four employees and work units without work environment data. We also excluded any quarters during which a work unit had not experienced stability or any of the three downsizing variables (i.e., quarters in which the units experience merger, spin-off, outsourcing, insourcing, or upsizing) [4]. This resulted in a sample consisting of 83,570 observations (employee-quarter), nested within 19,173 employees in 900 units.

Based on this sample, we also made a second dataset for analyses at the unit-level (see section on statistical analysis for details). This resulted in a sample of 2,500 observations (unit-quarter), nested within 876 units.

### Data

The present study employed two different sources of data drawn from registers in the human resources department (HR), and the health trust’s annual work environment survey. The data from the HR registers were merged at the individual level. The HR registers are continuously logged by the health trust, but for the purpose of analysis, the data were aggregated into the measurement unit employee-quarter, meaning one observation per employee per quarter. We aggregated the work environment survey to unit-level, so that each employee, in each quarter, was assigned the average value of control, role clarity and commitment for his or her work unit that year.

#### Unit-level downsizing and stability

We derived data on downsizing from health trust records of employment contracts. The register provides historical data regarding employees’ contracts throughout the study period. We were able to identify changes in staffing by tracking the specific work units’ employees in each quarter and their transfers between work units.

Unit-level downsizing was operationalized as the reduction of staff by at least 20% during a quarter, when the unit was not simultaneously experiencing a unit-level merger or unit-level outsourcing. The 20% cutoff is in line with the threshold used by Røed and Fevang [5] and Grønstad et al. [4]. We compared downsizing to stability.

We operationalized stability as a quarter during which the unit had not grown or been reduced in size by 20% or more. Moreover, stability entailed that at least 80% of the employees who worked in the unit during the previous quarter still worked in the unit at the end of the current quarter, and that at least 80% of the employees working in the unit at the end of the quarter also worked in the unit during the previous quarter. With this operationalization of stability, we also excluded quarters characterized by mergers, spin-offs, insourcing, upsizing, and outsourcing at the unit level, as well as a high degree of turnover. For a description of the excluded changes, their prevalence and relationship to sickness absence, please see Grønstad et al. [4].

For each quarter, the employees were coded as either anticipating downsizing in the upcoming quarter (i.e. “unit-level downsizing next quarter”), presently experiencing downsizing (i.e. “unit-level downsizing this quarter”), experienced downsizing in the previous quarter (i.e. “unit-level downsizing previous quarter”) or having stability.

#### Sickness absence

We derived data on sickness absence from the health trust’s register of absences. In the health trust records, each spell of absence was registered with a start and end date and the absence percentage. We aggregated the data to create two variables; whether each employee began a short spell of sickness absence (1–8 days) each quarter (1 = at least one short spell, 0 = no short spell), and whether an employee began a long spell of sickness absence ( > = 9 days) each quarter (1 = at least one long spell, 0 = no long spell). The cut-off point between short and long periods of absence (i.e. 9 days) was chosen because a medical certificate is only necessary for spells of absence lasting nine days or more. Spells of absence in which one spell started the day after another ended were merged. We included all periods of sickness absence irrespective of the percentage of absence (i.e. if an employee is 100% absent or partially absent). The health trust continuously records all sickness absence as this is a prerequisite for providing substitutes to units with absent personnel. The register can therefore be considered highly accurate and complete.

#### Work environment

We derived data on the work environment from the health trust’s annual work environment survey, conducted in 2011 (response rate 70%), 2012 (response rate 72%), 2013 (response rate 80%), and 2015 (response rate 76%). The annual work environment survey was conducted in the period September–October. Each survey was therefore merged with data from HR registers for the third and fourth quarters of the year. Given the timing of the work environment survey, we chose to exclude the first and second quarters in the present study.

The variables used in the current study were developed and validated as part of the General Nordic Questionnaire for Psychological and Social Factors at Work (QPS Nordic) [46]. All items were measured on a 5-point Likert scale ranging from “very seldom or never”/“disagree totally” to “very often or always”/“agree totally”. All items were measured on a 5-point Likert scale ranging from “very seldom or never”/“disagree totally” to “very often or always”/“agree totally”. The health trust utilized only some of the variables from QPS Nordic and only some scales of these variables. Because variables drawn from QPS Nordic have been rigorously validated, we decided to use these variables to ensure that our analyses rely on high-quality data.

Control at work was measured with two items from QPS Nordic [46], one pertaining to control over decisions (“Can you influence the amount of work assigned to you?”) and one pertaining to control of work pacing (“Can you set your own work pace?”). For control, Cronbach’s alpha was 0.795. Commitment to the organization was measured with two items from QPS Nordic [46] (“To my friends I praise this organization as a great place to work”, and “This organization really inspires me to give my very best job performance”). For commitment, Cronbach’s alpha was 0.874. Role clarity was measured using two items from QPS Nordic [46] (“Do you know what your responsibilities are?” and “Do you know exactly what is expected of you at work?”). For role clarity, Cronbach’s alpha was 0.832.

We conducted a Confirmatory factor analysis (CFA) using the SEM command in Stata. The model identified and did not require further adjustments to account for the two item factors. In two item factor models, the model may risk being under-identified if the factors are unrelated (or close to unrelated). The risk of under-identification is therefore larger when we use factor models with CFA with two items. While there are ways to address this challenge, under-identification was not a challenge in our CFA. The results are also in line with prior studies supporting the factor validity of items from QPS Nordic. The confirmatory factor analyses showed good psychometric properties for the items used; CFI was 0.998 and the root mean square error of approximation (RMSEA) was 0.027 (low 0.024 - high 0.031). According to Kelloway [70], RMSEA values below 0.10 constitute a good fit, whereas values below 0.05 are a particularly good fit to the data. Moreover, the result of Chi-Square was 160.517 and the Degrees of Freedom was 6. No challenges with convergent or discriminant validity were detected (using the condisc command in STATA). There were between 0.6 and 1.3% missing data on the items, and we used an Expectation-Maximization (EM) algorithm to handle the missing data.

For each year, the data from the work environment survey were aggregated to work-unit level and merged with the rest of the data. Due to anonymity concerns, we were only able to identify the units as opposed to individual respondents in the questionnaire. We were therefore unable to link the individual responses to the work environment questionnaire with the sickness absence data and aggregation of the work environment variables was therefore required. In other words, in the final analyses, employees’ individual sickness absence was predicted by their units’ mean response in the work environment survey, rather than their individual responses. Aggregated data, however, is also a strength as it allows us to focus on the general work environment in the unit rather than the different perceptions of individual employees. As argued by Hausknecht et al. [63], conceptualizations of sickness absence have often focused on individual-level predictors, thus diminishing the work-unit context that frames sickness absence behavior.

When aggregating the work environment survey, it was important to look at group agreement. In that regard, role clarity and commitment to the organization showed strong agreement within the work units, with an r_WG_ of 0.86 (role clarity) and 0.73 (commitment). Control showed only moderate agreement with an r_WG_ of 0.64 [71]. Traditionally, 0.7 has been used as a cut-off point denoting high interrater agreement and acceptable aggregation. However, LeBreton and Senter [71] argue that this cut-off point might be too high in some instances (e.g. the measure is not used for decisions involving specific individuals). Since we can only merge the work environment variables with the rest of the data at the work-unit level, we also aggregated the work environment factor control. However, the moderate level of agreement requires consideration when interpreting the findings.

#### Control variables

We included gender, salary, age, multiple jobs, temporary contracts, and occupation as control variables. All control variables were extracted from the HR registers. For unit-level analyses (with the work environment as outcome variable) the control variables were aggregated to the unit-level.

### Statistical analysis and study design

To investigate the three work environment factors as potential mediating variables in the relationship between unit-level downsizing and sickness absence, we conducted two sets of analyses, firstly by investigating the relationship between downsizing and the work environment (1) and secondly by investigating the relationship between downsizing, the work environment and sickness absence (2).

While simple mediation analysis can be conducted in one analysis by using structural equation modeling packages, the same result can be obtained by combining multiple regressions [72]. In this instance, conducting multiple analyses allowed us to benefit from using fixed effects analyses, as explained in more detail below. We calculated the size of the indirect effect by subtracting the direct effect from the total effect.

In the first set of analyses, we investigated downsizing as a potential explanatory variable for the work units’ average level of control, role clarity and commitment. The unit of measurement was unit-quarter.

We used fixed effects linear regression to investigate the relationship between each unit’s downsizing status during the third quarter of each year and the same unit’s work environment outcome during the same quarter. In this fixed-effects-design each work unit is only compared to itself at different times (e.g., as unit A changes downsizing status between 2011 and 2015, how does unit A’s average level of control, role clarity and commitment change in the same period). Because the analyses were conducted at the unit-level, all control variables were aggregated to unit-level (e.g. proportion of female employees at the unit).

We used unit-level data to avoid artificially inflating the results when analyzing a dependent variable which was constructed at the unit-level. For the same reason, we included only the third quarter each year for this set of analyses, as the outcome variable (the work environment factors) was only measured in the third quarter each year.

For the second set of analyses, we investigated downsizing and the work environment as potential explanatory variables for each employee’s risk of short and long-term sickness absence each quarter. Because sickness absence was measured at the individual level, the unit of measurement was employee-quarter. The outcome variable (sickness absence) and control variables were measured at the individual level (for each employee each quarter), but the explanatory variables (downsizing and the work environment) were measured at the unit-level (for each unit each quarter). We used fixed effects linear regression to investigate the relationship between each unit’s downsizing status and work environment variables during the third quarter and fourth quarter of each year and the individual employee’s sickness absence during the same quarter. In other words, we analyzed how sickness absence changed for each employee when they experienced downsizing, compared to when they experienced stability. The method ensured that the estimates were not a result of stable differences between employees working in downsizing units compared to employees working in stable units.

We used fixed effects linear probability models (LPM) to analyze the dichotomous outcome variable (i.e. sickness absence), which implies using linear regression with binary outcomes [73]. In logistic regression (or other methods where the variance of the error term is fixed), we cannot compare between models because the size of each coefficient depends on the amount of total explained variance within the model [73]. Instead, LPM is suggested as an alternative model with simulations demonstrating that the coefficients will be comparable to average marginal effects for logistic regression [73]. We therefore chose to use LPM since we were interested in comparing models (i.e. with and without the inclusion of the work environment variables). The analyses were performed in STATA15 using the xtreg command.

While all the analyses were executed using fixed effects, we also stress tested all analyses with a random effects structure equation modeling using gsem in Stata with random intercept at employee and work-unit level in order to ensure that no results were caused by dependency at multiple levels. Based on the gsem models, we estimated the significance of the indirect effects using the nlcom command.

## Results

The final sample consisted of 19,173 employees and 876 work units. Their demographics are presented in Table 1. A correlation matrix is enclosed in Table 2. In large organizations there will likely be instances where some units expand, some units downsize, and yet other units remain stable (i.e., no change) [4]. This was the case in the health trust being investigated in this paper.

**Table 1:** Descriptive statistics

		Mean	Percentage	Std. Dev
1	Downsizing next quarter	0,04		0,19
2	Downsizing this quarter	0,03		0,18
3	Downsizing previous quarter	0,01		0,12
4	Commitment	3,86		0,47
5	Control	2,84		0,61
6	Role Clarity	4,46		0,25
7	Short-term sickness absence	0,41	41	0,49
8	Long-term sickness absence	0,11	11	0,32
9	Female	0,74	74	0,44
10	Salary	524,63		237,59
11	Age	43,19		11,72
12	Multiple job holder	0,17	17	0,38
13	Temporary contract	0,17	17	0,37
14	Physician	0,11	11	0,31
15	Other patient-related position	0,24	24	0,43
16	Administration/management	0,15	15	0,36
17	Kitchen/cleaning/orderly	0,05	5	0,21
18	Other operations	0,04	4	0,21
19	Other	0,03	3	0,18

N observations 83,570

**Table 2:** Correlation matrix

	Variable	Correlation matrix																																			
		1		2		3		4		5		6		7		8		9		10		11		12		13		14		15		16		17		18	
1	Downsizing next quarter	1.00																																			
2	Downsizing this quarter	−0.04	*	1.00																																	
3	Downsizing previous quarter	−0.02	*	−0.02	*	1.00																															
4	Commitment	−0.03	*	−0.03	*	−0.05	*	1.00																													
5	Control	−0.03	*	−0.04	*	−0.02	*	0.24	*	1.00																											
6	Role Clarity	−0.06	*	−0.04	*	−0.02	*	0.55	*	0.04	*	1.00																									
7	Short-term sickness absence	−0.04	*	0.00		0.01	*	0.00		−0.04	*	0.04	*	1.00																							
8	Long-term sickness absence	−0.01	*	−0.01	*	0.01	*	−0.01	*	−0.01	*	0.02	*	0.05	*	1.00																					
9	Female	−0.03	*	−0.01	*	0.00		0.07	*	0.00		0.08	*	0.06	*	0.06	*	1.00																			
10	Salary	0.10	*	0.05	*	0.00		−0.05	*	−0.04	*	−0.09	*	−0.15	*	−0.08	*	−0.10	*	1.00																	
11	Age	−0.02	*	−0.02	*	0.00		−0.07	*	0.03	*	0.03	*	−0.07	*	0.01	*	−0.10	*	0.09	*	1.00															
12	Multiple job holder	0.07	*	0.03	*	0.01	*	−0.01		−0.07	*	−0.03	*	−0.03	*	−0.04		0.00	*	0.65	*	−0.15	*	1.00													
13	Temporary contracts	0.12	*	0.09	*	0.01		0.00		0.02	*	−0.09	*	−0.06	*	−0.06	*	0.01	*	0.15	*	−0.36	*	0.20	*	1.00											
14	Physician	0.18	*	0.15	*	0.01	*	−0.18	*	−0.18	*	−0.17	*	−0.15	*	−0.07	*	−0.19	*	0.42	*	0.05	*	0.15	*	0.29	*	1.00									
15	Other patient-related position	−0.02	*	−0.02	*	0.00		−0.05	*	−0.06	*	0.04	*	0.06	*	0.02	*	0.01	*	−0.20	*	0.00		−0.09	*	−0.10	*	−0.19	*	1.00							
16	Administration/management	0.01		−0.01	*	−0.01	*	−0.07	*	0.13	*	−0.04	*	−0.04	*	−0.01	*	0.02		0.02	*	0.17	*	−0.07	*	−0.12	*	−0.15	*	−0.24	*	1.00					
17	Kitchen/cleaning/orderly	0.00		0.00		0.04	*	0.04	*	0.13	*	0.06	*	0.08	*	0.08	*	−0.15	*	−0.17	*	0.07	*	−0.07	*	−0.07	*	−0.08	*	−0.13	*	−0.10	*	1.00			
18	Other operations	−0.01	*	−0.03	*	−0.01	*	−0.02	*	0.15	*	−0.03	*	−0.02	*	0.00	*	−0.16		−0.09	*	0.04	*	−0.06	*	−0.04	*	−0.07	*	−0.12	*	−0.09	*	−0.05	*	1	
19	Other	0.01		0.00		−0.01	*	0.04	*	0.31	*	−0.05	*	−0.11	*	−0.04	*	−0.04	*	0.06	*	−0.07	*	0.00		0.24	*	−0.06	*	−0.10	*	−0.08	*	−0.04	*	−0.04	*

*N*=83,570 observations

*p>0.05 (correlation matrix is not adjusted for dependency in the data)

### Downsizing and the work environment

Table 3 presents the results of the analyses of the relationship between unit-level downsizing and the three unit-level work environment factors; control (Model 1), role clarity (Model 2), and commitment (Model 3). The analyses include 2,500 observations (work unit-quarter), nested within 876 work units. Each unit is observed once a year (i.e. the third quarter) for a period of up to four years. The results show that the work units scored significantly lower on commitment in the quarter during (−0.24 *p* < 0.001) and after unit-level downsizing (−0.23 *p* < 0.05). Similarly, work units scored significantly lower on control in the quarter after unit-level downsizing (−0.28 *p* < 0.01). No significant difference was found for role clarity.

**Table 3:** A fixed effects linear analysis of the relationship between unit-level downsizing and unit-level control, role clarity and commitment

		95% CI			95% CI			95% CI	
A work environment for:	Control	LL	UL	Role Clarity	LL	UL	Commitment	LL	UL
Downsizing next quarter	−0.047	[−0.107,0.012]		0.015	[−0.031,0.060]		0.056	[−0.019,0.132]	
Downsizing this quarter	−0.078	[−0.173,0.016]		−0.067	[−0.139,0.005]		−0.238***	[−0.357,−0.118]	
Downsizing previous quarter	−0.276**	[−0.446,−0.105]		0.043	[−0.087,0.173]		−0.225*	[−0.441,−0.008]	
**Control variables: **									
No. of employees	−0.002	[−0.005,0.000]		−0.001	[−0.003,0.001]		−0.003	[−0.006,0.000]	
Female	−0.199	[−0.416,0.017]		−0.067	[−0.231,0.098]		−0.034	[−0.308,0.240]	
Salary	0	[−0.000,0.001]		0.000**	[0.000,0.001]		0.001***	[0.001,0.001]	
Age	−0.001	[−0.009,0.007]		0.004	[−0.002,0.010]		−0.004	[−0.014,0.006]	
Multiple job holder	−0.076	[−0.227,0.074]		−0.243***	[−0.357,−0.128]		−0.615***	[−0.806,−0.425]	
Temporary contracts	−0.042	[−0.232,0.148]		−0.009	[−0.153,0.136]		0.058	[−0.182,0.299]	
Physician	0.131	[−0.325,0.586]		−0.082	[−0.428,0.265]		−0.026	[−0.603,0.551]	
Other patient-related position	0.15	[−0.232,0.532]		−0.457**	[−0.748,−0.167]		−0.156	[−0.640,0.327]	
Administration/management	0.559**	[0.175,0.943]		−0.344*	[−0.636,−0.051]		−0.11	[−0.596,0.376]	
Kitchen/cleaning/orderly	0.583*	[0.027,1.140]		−0.218	[−0.642,0.205]		0.168	[−0.536,0.873]	
Other operations	0.536*	[0.079,0.993]		−0.430*	[−0.778,−0.083]		0.037	[−0.542,0.616]	
Other	0.528	[−0.025,1.081]		−0.262	[−0.682,0.159]		0.266	[−0.434,0.966]	
_cons	2.845***	[2.356,3.333]		4.468***	[4.097,4.840]		3.665***	[3.047,4.284]	
	Variance			Variance			Variance		
sigma_u	0.583			0.316			0.488		
sigma_e	0.260			0.198			0.329		
rho	0.834			0.718			0.689		

N work units: 876; N observations: 2,500

95% confidence intervals in brackets

Control: includes the relationship between downsizing and control at the unit-level

Role Clarity: includes the relationship between downsizing and role clarity at the unit-level

Commitment: includes the relationship between downsizing and commitment at the unit-level

All control variables are aggregated at the unit-level

*p<0.05

**p<0.01

***p<0.001

### Downsizing, the work environment and short-term sickness absence

Table 4 presents the results of the analyses of each employees’ risk of short-term sickness absence each quarter. The analyses include 83,570 observations (employee-quarter), nested within 19,173 employees. The first analysis (Model 0) includes unit-level downsizing (next quarter, this quarter, and previous quarter) as the explanatory variable, in addition to the control variables. The following analyses also include unit-level commitment (Model 1), control (Model 2), role clarity (Model 3) and all three work environment factors (Model 4).

**Table 4:** A fixed effects linear probability model of the relationship between downsizing and short-term sickness absence, without and with control for work environment factors

	Model 0		Model 1		Model 2		Model 3		Model 4	
Downsizing next quarter	−0.045***	[−0.066,−0.024]	−0.045***	[−0.066,−0.024]	−0.045***	[−0.066,−0.024]	−0.045***	[−0.066,−0.024]	−0.045***	[−0.066,−0.024]
Downsizing this quarter	0.034**	[0.013,0.056]	0.034**	[0.013,0.055]	0.034**	[0.013,0.056]	0.034**	[0.013,0.056]	0.034**	[0.013,0.056]
Downsizing previous quarter	0.049**	[0.020,0.079]	0.047**	[0.018,0.077]	0.049**	[0.020,0.078]	0.049**	[0.020,0.079]	0.047**	[0.018,0.077]
**Work environment:**										
Commitment			−0.026***	[−0.038,−0.013]					−0.032***	[−0.047,−0.017]
Control					−0,01	[−0.026,0.007]			−0,001	[−0.018,0.016]
Role Clarity							−0,004	[−0.026,0.018]	0,023	[−0.002,0.048]
**Control variables:**										
Salary	−0.000***	[−0.000,−0.000]	−0.000**	[−0.000,−0.000]	−0.000***	[−0.000,−0.000]	−0.000***	[−0.000,−0.000]	−0.000**	[−0.000,−0.000]
Multiple job holder	0,003	[−0.018,0.024]	−0,001	[−0.022,0.020]	0,003	[−0.018,0.024]	0,003	[−0.018,0.024]	0	[−0.021,0.021]
Temporary contracts	−0,003	[−0.021,0.016]	−0,005	[−0.024,0.013]	−0,003	[−0.022,0.016]	−0,003	[−0.022,0.016]	−0,005	[−0.024,0.013]
_cons	0.450***	[0.430,0.470]	0.546***	[0.495,0.598]	0.477***	[0.427,0.526]	0.468***	[0.368,0.567]	0.471***	[0.368,0.573]
Random-effects Parameters										
	Variance		Variance		Variance		Variance		Variance	
sigma_u	0,349		0,349		0,349		0,349		0,349	
sigma_e	0,426		0,426		0,426		0,426		0,426	
rho	0,402		0,403		0,402		0,402		0,403	

N employees: 19,173; N observations: 83,570

95% confidence intervals in brackets

Model 0: shows the relationship between downsizing and sickness absence, including control variables

Model 1: Model 0 + control for commitment

Model 2: Model 0 + control for control

Model 3: Model 0 + control for role clarity

Model 4: Model 0+ all three work environment variables (Models 1,2 and 3)

*p<0.05

**p<0.01

***p<0.001

The results show that employees’ probability of short-term sickness absence is significantly lower in the quarter preceding unit-level downsizing (−0.05 *p* < 0.001) and significantly higher during unit-level downsizing (0.03 *p* < 0.01), and the quarter following unit-level downsizing (0.05 *p* < 0.01). We used linear probability models (LPM) to analyze the dichotomous outcome variable (i.e. sickness absence), and the results should therefore be read as marginal effects. A coefficient of 0.05 equals an estimated increase of 5% points. The results further show that when the level of commitment was higher in the work unit, the employees’ probability of short-term sickness absence was significantly lower (commitment: −0.03 *p* < 0.001).

A comparison of the relationship between downsizing and sickness absence before and after the inclusion of the work environment factors only produced minor differences in magnitude. The relationship between downsizing in the previous quarter and short-term sickness absence changed from 0.049 to 0.047 after the inclusion of commitment, and it remained at 0.047 after the inclusion of all three work environment factors. In other words, the results indicate that the probability of a period of short-term sickness absence is 4.9% points higher in the quarter after downsizing than in quarters with stability. Lower levels of commitment may account for 4% of this increase.

### Downsizing, the work environment and long-term sickness absence

Table 5 presents the results of the analysis of each employee’s risk of long-term sickness absence each quarter. As in Table 4, the analysis includes 83,570 observations (employee-quarter), nested within 19,173 employees.

**Table 5:** A fixed effects linear probability model of the relationship between downsizing and long-term sickness absence, without and with control for work environment factors

	Model 0		Model 1		Model 2		Model 3		Model 4	
Downsizing next quarter	−0,004	[−0.019,0.010]	−0,005	[−0.019,0.010]	−0,005	[−0.020,0.010]	−0,004	[−0.019,0.010]	−0,005	[−0.020,0.010]
Downsizing this quarter	−0,004	[−0.019,0.011]	−0,004	[−0.019,0.011]	−0,004	[−0.019,0.011]	−0,004	[−0.019,0.011]	−0,004	[−0.019,0.011]
Downsizing previous quarter	0,02	[−0.000,0.041]	0,02	[−0.001,0.041]	0,02	[−0.001,0.041]	0,02	[−0.000,0.041]	0,02	[−0.001,0.041]
**Work environment:**										
Commitment			−0,002	[−0.011,0.007]					0,001	[−0.009,0.012]
Control					−0.012*	[−0.023,−0.001]			−0,012	[−0.024,0.000]
Role Clarity							−0,005	[−0.021,0.010]	−0,003	[−0.020,0.015]
**Control variables:**										
Salary	0	[−0.000,0.000]	0	[−0.000,0.000]	0	[−0.000,0.000]	0	[−0.000,0.000]	0	[−0.000,0.000]
Multiple job holder	−0,011	[−0.025,0.004]	−0,011	[−0.026,0.004]	−0,011	[−0.025,0.004]	−0,011	[−0.026,0.004]	−0,011	[−0.026,0.004]
Temporary contracts	−0.041***	[−0.054,−0.028]	−0.041***	[−0.054,−0.028]	−0.041***	[−0.054,−0.028]	−0.041***	[−0.054,−0.028]	−0.041***	[−0.054,−0.028]
_cons	0.125***	[0.111,0.139]	0.134***	[0.097,0.170]	0.158***	[0.123,0.193]	0.148***	[0.078,0.217]	0.165***	[0.093,0.237]
	Variance		Variance		Variance		Variance		Variance	
sigma_u	0,207		0,207		0,207		0,207		0,207	
sigma_e	0,298		0,298		0,298		0,298		0,298	
rho	0,325		0,325		0,325		0,325		0,325	

N employees 19,173; N observations: 83,570

95% confidence intervals in brackets

Model 0: shows the relationship between downsizing and sickness absence, including control variables

Model 1: Model 0 + control for commitment

Model 2: Model 0 + control for control

Model 3: Model 0 + control for role clarity

Model 4: Model 0+ all three work environment variables (Models 1,2 and 3)

*p<0.05

**p<0.01

***p<0.001

The results show no relationship between unit-level downsizing and long-term sickness absence. Employees working in units with higher control also had a lower risk of long-term sickness absence (control: −0.01 *p* < 0.05). No significant effect was found for role clarity or commitment.

### The robustness of the findings

To test the robustness of the findings, all results were stress-tested using random effects structural equation modeling (Table A supplementary material). All relationships remained significant and in the same direction. Furthermore, the indirect effects between downsizing and sickness absence via control and commitment were significant.

## Discussion

Despite the many studies focusing on the relationship between organizational change, health and sickness absence, few studies have empirically investigated why organizational change affects health and sickness absence [19]. Moreover, those studies that have investigated mediation have only included a handful of the same work environment factors. This study contributes to our theoretical understanding of the relationship by empirically investigating potential linking mechanisms, including two work environment factors that have not previously been investigated.

In line with our previous research [4], the study demonstrates that different phases of downsizing can relate differently to employees’ sickness absence. The results showed an increased risk of short-term sickness absence in the quarter during and after unit-level downsizing. The findings are in general congruence with previous studies showing that downsizing may lead to adverse health effects and increased sickness absence [5, 7, 8, 11]. However, notably, and in line with Østhus and Mastekaasa [30], the results are not significant for long-term absence.

The results also showed reduced odds of sickness absence in the quarter before unit-level downsizing. The reduction in sickness absence prior to the change may be explained by employees’ perceptions of increased job insecurity. However, it is also possible that the employees feel a need to participate in and influence the change process and therefore attend work. The increased odds of sickness absence during and after downsizing is often explained by adverse changes in the work environment suggesting that certain types of organizational change, such as downsizing, adversely affect employees’ health and subsequent sickness absence through negative alterations in the work environment [18]. Our results partially support this argument.

In line with Hypothesis 1, the reported level of control in the work units significantly dropped compared to during periods of stability after unit-level downsizing. Moreover, when levels of control in the work unit were reduced, employees’ risk of long-term sickness absence increased. This finding is in line with previous literature that highlights control as an important work-related factor that influences employees’ health and sickness absence [18, 37, 40, 43]. However, control was not related to short-term sickness absence, and an inspection of the potential mediating relationship indicated that including control in the analyses did not alter the relationship between downsizing and any measure of sickness absence. In contrast, Kivimäki et al. [18] found that a reduction in skill discretion could explain 12% of the increased risk of long-term sickness absence during major organization-wide downsizing. One possible reason for the limited importance of control as a mediating variable in the current study could be the health trust context. In a health trust setting, employees may perceive fluctuating patient needs and best evidence practice as legitimate and important reasons for having a low level of control. Indeed, the employee group reporting the lowest levels of control was physicians, followed by nurses and other patient-oriented personnel. It is also possible that control is primarily an individual experience rather than a quality of the work unit, and the results indeed showed a lower level of group agreement on control than is ideally recommended for aggregation. Nonetheless, the substantial magnitude of control as a mediator between organizational change and sickness absence identified by Kivimäki et al. [18] has not been replicated in later studies. Future studies should investigate to what extent and when loss of control is an important cause of reduced health and increased absence during organizational change.

Contrary to Hypothesis 2, unit-level downsizing was not significantly related to role clarity. Nor, unexpectedly, was role clarity related to a reduced risk of absence. This contrasts with prior research linking role clarity to improved health and reduced absence [37, 48–50]. Though, some previous studies have yielded results that could reflect that the consequences of role clarity are complex, possibly nonlinear, or different in different populations. More specifically, Sundstrup et al. [51] found that medium levels of role clarity were related to a greater risk of long-term sickness absence, while this was not true for low levels of role clarity. Väänänen et al. [74] only found a relationship between role clarity and sickness absence among white-collar men, and not among blue-collar men or among women. In the current sample, the highest levels of role clarity were found among women, and specifically among kitchen and cleaning personnel.

In line with Hypothesis 3a, we found significantly lower levels of organizational commitment in the work units during and after unit-level downsizing. These results support the argument that organizational changes such as downsizing have the potential to undermine employee commitment [67, 75]. Indeed, downsizing may influence the bond between the organization and the individual employees by altering valued aspects of work and the organization, and by violating the reciprocal nature of the employer-employee relationship [66]. The results are also in congruence with previous results showing lower levels of organizational commitment among downsizing survivors than among workers unaffected by downsizing [67]. By using a longitudinal dataset, however, our results show that such differences are not caused by stable differences between employees who are and are not exposed to downsizing (e.g., if organizations with less committed employees needed to downsize more frequently due to being less profitable).

Moreover, and in line with Hypothesis 3b, reduced organizational commitment at the work unit was significantly related to a greater risk of short-term sickness absence. By analyzing the magnitude of the indirect relationship between unit-level downsizing and sickness absence, organizational commitment was found to explain approximately 4% of the increased risk of short-term sickness absence in the quarter after unit-level downsizing. Reduced commitment may contribute to increasing the risk of sickness absence by affecting both employee health and attendance motivation. On the one hand, organizational commitment can be beneficial for employee health by reinforcing perceptions of belongingness and security, and preventing job displeasure [59, 60], while on the other, commitment can also foster attendance motivation and potentially encourage employees to attend work and refrain from being absent because of minor health complaints, i.e., sickness presence [76]. Short-term sickness absence is, to a greater extent than long-term sickness absence, influenced by factors other than health [77]. Granted that unit-level downsizing and organizational commitment are most robustly related to short-term sickness absence in the present study, the question is raised of whether at least part of the increase in absence detected is due to weaker attendance motivation after downsizing rather than reduced health. This does not, however, imply that employees on sick leave are not necessarily ill, but rather that their probability of attending work despite illness is reduced.

In sum, our results indicate that unit-level downsizing can lead to an increased risk of short-term sickness absence. Adverse alterations in the work environment caused by the change, particularly reduced levels of organizational commitment in the work units, contribute to explaining this relationship. However, the work environment factors investigated in this paper only explain a small part of the relationship between unit-level downsizing and sickness absence. Combined, the three work environment factors accounted for a total of 4% of the increase in short-term sickness absence in the quarter after unit-level downsizing.

The small amount of variance explained by organizational commitment in our study does not necessarily mean that commitment is irrelevant to understanding the relationship between organizational change and sickness absence. The results could imply that organizational change and sickness absence are interrelated via a wide variety of mechanisms. As discussed by Zapf et al. [78], small correlations are not necessarily a weakness but can simply mean that there is a multifactorial relationship between workplace events and employee health. Other studies have previously highlighted the variety of variables involved in explaining work-related health impairments and sickness absence [79, 80]. As such, this study helps to untangle the complexity by introducing and testing two potential mediators of the relationship between unit-level downsizing and sickness absence that have so far received less attention (i.e., organizational commitment and role clarity). Nonetheless, given the low percentage of explained variance in the present study, it is pertinent to question whether other work environment factors would have been more relevant to include. As the focus of this paper is downsizing, it is possible that it could be argued that the most prominent cause of increased sickness absence is stress due to increased job insecurity. Indeed, earlier research often argues that perceptions of job insecurity increase in times of organizational change, in particular related to downsizing [3, 14, 18]. However, using the same data material as the present paper, our previous study found that permanent employees demonstrated an increase in sickness absence similar to that of temporary employees [81]. Within the Norwegian healthcare setting, downsizing very rarely leads to termination of permanent employment contracts. Instead, healthcare organizations downsize through alternative means such as by not renewing temporary contracts. If job insecurity had been the dominant cause of increased sickness absence, the increase should have been substantially greater among temporary employees. Collectively, the present findings and existing literature highlight that future studies should consider a broader range of potential mediators for the relationship between organizational change and health consequences and absence.

### Strengths and limitations

A primary strength of this study is the longitudinal design that relies on three separate and different sources of data, including objective records of organizational change and sickness absence. The data and study design therefore limit the risk of biases such as same-source bias [82]. Furthermore, the data enabled the use of advanced statistical methods, thereby reducing the risk of finding spurious correlations (e.g., by allowing us to establish that the level of commitment in a work unit is reduced after unit-level downsizing, as compared to only establishing that downsized units also tend to have poorer levels of commitment). All work environment variables were aggregated to the unit level. As aggregation entails a reduction of data, we risk undermining the possibility that perceptions of the work environment may vary substantially between employees in the same unit. This is likely particularly true for the measure of control, which did not show ideal group agreement for aggregation. Aggregation also means that we obtain limited insights into the individual employees’ subjective experiences of downsizing and the work environment. Such insights arguably hold value for arriving at a better understanding of the psychological mechanisms explaining the relationship between downsizing and sickness absence. Indeed, if relevant individual variation in the data remains unaccounted for, the significance of the studied factors could be underestimated. Despite fewer observations, aggregation also has clear advantages as it reduced the chance of having biases and risks emerging from individual predispositions influencing the data. Furthermore, the unit-level approach permitted the investigation of the work groups’ work environment, which is beneficial because earlier research on health and sickness absence has noted that limited attention to group-level variables may lead to inadequate conceptualizations of ailments [83, 84]. Yet, research on how group-level variables influence individual-level outcomes, such as sickness absence, is still pending [84]. By aggregating the data to the work unit, we thus focused on the qualities of the work unit and not of the individual employee. Hence, managers and HR practitioners may find aggregated information particularly useful [85], because it provides a lens into mechanisms explaining why a particular result plays out. Still, future studies should attempt to use multilevel models that include both unit and individual observations of the work environment and thus provide a more nuanced understanding of the relationship between downsizing and sickness absence at both levels. Using such models would have enabled us to investigate individual-level predictors of sickness absence while at the same time considering the unit-level structure of the data. This could potentially have generated a clearer view of the overall relationship but was not possible in the present study.

A related limitation of the current study is the lack of data pertaining to the employees’ perception of the unit-level downsizing. We have operationalized unit-level downsizing as a reduction of personnel of at least 20% in the work unit. In the health trust setting, these reductions generally occur without lay-offs. Moreover, downsized employees will often continue to work at the health trust, but in another unit. It is therefore possible that employees perceive some of the changes we have categorized as unit-level downsizing, as reorganization. This is important because the consequences of downsizing for the work environment and health are not necessarily the same as those of reorganization [14].

It is also worth noting that we did not utilize the full validated scales for the three work environment variables. Utilizing preexisting data, the scales were measured with two items, based on the items included in the work environment survey.

We also recognize that while the paper utilizes complete data on sickness absence, there may be missing data or non-response in the work environment data. As the data were aggregated, however, the risk of bias related to non-response at the individual level is reduced. Some units were also excluded entirely from the data because of non-participation in the survey. The response rate was generally high, and the number of units excluded because of non-participation in the work environment survey was low. This was typically the case for units with few employees.

The paper focuses on the healthcare sector. The healthcare sector employs around one fifth of the workers in Norway [24], and understanding the mechanism of change within this sector is therefore important. At the same time, it is relevant to acknowledge that focusing on only one sector might limit the generalizability of the findings to other sectors. The public and state-owned nature of the health trust suggests it might differ from private equivalents. In addition, the healthcare sector suffers from manpower shortages [86]. The effects of downsizing might therefore be experienced differently in sectors where manpower is less in demand.

A final challenge of the current study is the presence of multiple testing. We have analyzed several different relationships between three stages of downsizing, included three different work environment factors and two sickness absence measures. The use of multiple testing increases the probability of finding significant effects by chance [87]. This needs to be taken into consideration when reading and interpreting the results.

### Practical implications

The results of this paper offer recommendations for managers and HR practitioners who contribute to or oversee the implementation of organizational changes. Gaining a broader understanding of the mechanisms that drive, or derail change implementation constitutes an important knowledge bank for those tasked with change implementation. Getting access to such knowledge can help decision-makers and HR representatives to source the right resources and capabilities for implementation. To this end, knowledge of mediators can be a powerful tool to improve and govern practitioners’ delivery of change. The result showing that some of the increase in sickness absence can be explained by lower commitment following change indicates that managers gain a knowledge-based point of departure from which the employees’ tolerance for change can be increased by reinforcing a work environment aiming to enhance organizational commitment. Obtaining such insights from a mediation analysis allows practitioners to get closer to important aspects in the chain of events and be especially aware of them during change. In this way, organizational policies can be better tailored to reinforce important aspects that boost employee commitment. This kind of knowledge is especially important with regard to developing sound and reliable interventions and measures for smooth change implementation and can support management efforts to make taking care of employees’ health a key element in any organization’s business strategy.

## Conclusion

In line with previous studies, the current study supports that the work environment at least partially mediates the relationship between organizational change and sickness absence. Moreover, the study contributes to the field by establishing that organizational commitment is a relevant factor in understanding the relationship between unit-level downsizing and sickness absence. However, the collective evidence also shows that there is a clear need to explore and test additional factors that could further contribute to explaining the complexity of the relationship between organizational change, health, and sickness absence.

## Supplementary Information

Supplementary Material 1.

Supplementary Material 2.

## Data Availability

In regard to data sharing; the data are the property of the hospital. Upon reasonable request we will facilitate contact with the participating hospital.

## Abbreviations

HR: Human Resources
QPS (Nordic): General Nordic Questionnaire for Psychological and Social Factors at Work
SEM: Structural Equation Model
CFA: Confirmatory Factor Analysis
RMSEA: Root Mean Square Error of Approximation
EM (algorithm): Expectation-Maximization algorithm
LPM: Linear Probability Model

## References

[1] Brown C, Arnetz B, Petersson O. Downsizing within a hospital: cutting care or just costs? Soc Sci Med. 2003;57(9):1539–46. 12948565 10.1016/s0277-9536(02)00556-7

[2] Schulz A-C, Wiersema MF. The impact of earning expectations of corporate downsizing. Strateg Manag J. 2018;39(10):2691–702.

[3] Kivimäki M, Vahtera J, Pentti J, Thomson L, Griffiths A, Cox T. Downsizing, changes in work, and self-rated health of employees: A 7-year 3-wave panel study. Anxiety Stress Coping. 2001;14(1):59–73.

[4] Grønstad A, Kjekshus LE, Tjerbo T et al. Organizational change and the risk of sickness absence: a longitudinal multilevel analysis of organizational unit-level change in hospitals. BMC Health Serv Res. 2019;19:1–11. 10.1186/s12913-019-4745-2 PMC6880570 31771576

[5] Røed K, Fevang E. Organizational change, absenteeism, and welfare dependency. J Hum Resour. 2007;42(1):156–93.

[6] Harney B, Fu N, Freeney Y. Balancing tensions: buffering the impact of organisational restructuring and downsizing on employee well-being. Hum Resource Manage J. 2018;28(2):235–54.

[7] Andreeva E, Hanson LLM, Westerlund H, Theorell T, Brenner MH. Depressive symptoms as a cause and effect of job loss in men and women: evidence in the context of organisational downsizing from the Swedish longitudinal occupational survey of health. BMC Public Health. 2015;15:1045–56. 26458894 10.1186/s12889-015-2377-y PMC4603822

[8] Dragano N, Verde PE, Siegrist J. Organisational downsizing and work stress: testing synergistic health effects in employed men and women. J Epidemiol Community Health. 2005;59:694–9. 16020648 10.1136/jech.2005.035089 PMC1733120

[9] Grunberg L, Moore SY, Greenberg E. Differences in psychological and physical health among layoff survivors: the effect of layoff contact. J Occup Health Psychol. 2001;6:15–25. 11199253 10.1037//1076-8998.6.1.15

[10] Modrek S, Cullen MR. Health consequences of the ‘great recession’ on the employed: evidence from an industrial cohort in aluminum manufacturing. Soc Sci Med. 2013;92:105–13. 23849284 10.1016/j.socscimed.2013.04.027 PMC3783210

[11] Kaspersen SLPK, Carlsen F, Ose SO, Bjorngaard JH. Employees’ drug purchases before and after organizational downsizing: a natural experiment on the Norwegian working population (2004–2012). Scand J Work Environ Health. 2012;43(4):307–15. 10.5271/sjweh.3637 28350411

[12] Claussen BNØ, Reime LJ, Leyland AH. Proof firm downsizing and diagnosis-specific disability pensioning in Norway. BMC Public Health. 2013;13:1–9. 10.1186/1471-2458-13-27 PMC3655911 23311568

[13] Ahammer AG, Winter-Ebmer D. The health externalities of downsizing. CINCH series, no 2021/02. Essen: University of Duisburg-Essen, CINCH - Health Economics Research Center; 2021.

[14] Østhus S. For better or worse? Workplace changes and the health and well-being of Norwegian workers. Work Employ Soc. 2007;21(4):731–50.

[15] Østhus S. Health effects of downsizing survival and job loss in Norway. Soc Sci Med. 2012;75(5):946–53. 22682662 10.1016/j.socscimed.2012.04.036

[16] Jensen JH, Flachs EM, Skakon J, Rod NH, Bonde JP. Dual impact of organisational change on subsequent exit from work unit and sickness absence: a longitudinal study among public healthcare employees. Occup Environ Med. 2018;75(7):479–85. 29760173 10.1136/oemed-2017-104865 PMC6035486

[17] Robinson O, Griffiths A. Coping with the stress of transformational change in a government department. J Appl Behav Sci. 2005;41(2):204–21.

[18] Kivimäki M, Vahtera J, Pentti J, Ferrie JE. Factors underlying the effect of organisational downsizing on health of employees: longitudinal cohort study. BMJ. 2000;320(7240):971–5. 10753148 10.1136/bmj.320.7240.971 PMC27336

[19] Grønstad AF. Exploring work-related attributions of sickness absence during organizational change. Int J Workplace Health Manage. 2017;10(3):192–212.

[20] López Bohle SA, Chambel MJ, Diaz-Valdes Iriarte A. Job insecurity, procedural justice and downsizing survivor affects. Int J Hum Resource Manage. 2018;32(3):1–20.

[21] Fløvik L, Knardahl S, Christensen JO. Organizational change and employee mental health: A prospective multilevel study of the associations between organizational changes and clinically relevant mental distress. Scand J Work Environ Health. 2019;45(2):134. 10.5271/sjweh.3777 30334062

[22] Geuskens GA, Koppes LL, van den Bossche SN, Joling CI. Enterprise restructuring and the health of employees: a cohort study. J Occup Environ Med. 2012;54(1):4–9. 22157732 10.1097/JOM.0b013e31823c766e

[23] Eriksson U-B, Starrin B, Janson S. Long-Term sickness absence due to burnout: absentees’ experiences. Qual Health Res. 2008;18(5):620–32. 18420536 10.1177/1049732308316024

[24] OECD. Health at a glance. OECD indicators. Paris: OECD Publishing; 2019. 10.1787/4dd50c09-en.

[25] Hagen TP, Kaarbøe OM. The Norwegian hospital reform of 2002: central government takes over ownership of public hospitals. Health Policy. 2006;76(3):320–33. 16099530 10.1016/j.healthpol.2005.06.014

[26] Kjekshus LE, Bernstrøm VH, Dahl E, Lorentzen T. The effect of hospital mergers on long-term sickness absence among hospital employees: a fixed effects multivariate regression analysis using panel data. BMC Health Serv Res. 2014;14(1):50. 24490750 10.1186/1472-6963-14-50 PMC3922609

[27] Bernstrom VH, Kjekshus LE. Effect of organisational change type and frequency on long-term sickness absence in hospitals. J Nurs Manag. 2015;23(6):813–22. 24612363 10.1111/jonm.12218

[28] Ingelsrud MH. Reorganization increases long-term sickness absence at all levels of hospital staff: panel data analysis of employees of Norwegian public hospitals. BMC Health Serv Res. 2014;14(1):411. 25239190 10.1186/1472-6963-14-411 PMC4177695

[29] Koivunen N, Viitala R, Ekman K. How managers experience downsizing: navigating among professional, loyal, empathic, and critical positions. J Change Manage. 2024;24(4):301–24.

[30] Østhus S, Mastekaasa A. The impact of downsizing on remaining workers’ sickness absence. Soc Sci Med. 2010;71(8):1455–62. 20728975 10.1016/j.socscimed.2010.06.045

[31] Burke RJ, Greenglass ER. Effects of hospital restructuring on full time and part time nursing staff in Ontario. Int J Nurs Stud. 2000;37(2):163–71. 10684958 10.1016/s0020-7489(99)00058-9

[32] Westerlund H, Ferrie J, Hagberg J, Jeding K, Oxenstierna G, Theorell T. Workplace expansion, long-term sickness absence, and hospital admission. Lancet. 2004;363(9416):1193–7. 15081652 10.1016/S0140-6736(04)15949-7

[33] Saksvik PØ, Tvedt SD, Nytrø K, Andersen GR, Andersen TK, Buvik MP, Torvatn H. Developing criteria for healthy organizational change. Work Stress. 2007;21(3):243–63.

[34] Breinegaard N, Jensen JH, Bonde JP, Breinegaard N. Organizational change, psychosocial work environment, and non-disability early retirement: a prospective study among senior public employees. Scand J Work Environ Health. 2017;43(3):234–40. 28166362 10.5271/sjweh.3624

[35] Bernstrøm VH, Kjekshus LE. Effect of organisational change type and frequency on long-term sickness absence in hospitals. J Nurs Manag. 2015;23(6):813-22. 10.1111/jonm.12218 24612363

[36] Karasek R, Theorell T. Healthy work: stress, productivity, and the reconstruction of working life. New York: Basic Books; 1990.

[37] Roelen C, Koopmans P, Bültmann U, Groothoff J, Klink J. Psychosocial work conditions and registered sickness absence: a 3-year prospective cohort study among office employees. Int Arch Occup Environ Health. 2009;82(9):1107–13. 19471954 10.1007/s00420-009-0425-6 PMC2746900

[38] Head J, Kivimaki M, Martikainen P, Vahtera J, Ferrie JE, Marmot MG. Influence of change in psychosocial work characteristics on sickness absence: the Whitehall II study. J Epidemiol Community Health. 2006;60(1):55–61. 16361455 10.1136/jech.2005.038752 PMC2465520

[39] Karasek RA. Job demands, job decision latitude, and mental strain: implications for job redesign. Adm Sci Q. 1979;24(2):285–308.

[40] Marmot MG, Bosma H, Hemingway H, Brunner E, Stansfeld S. Contribution of job control and other risk factors to social variations in coronary heart disease incidence. Lancet. 1997;350(9073):235–9. 9242799 10.1016/s0140-6736(97)04244-x

[41] Bordia P, Hobman E, Jones E, Gallois C, Callan VJ. Uncertainty during organizational change: types, consequences, and management strategies. J Bus Psychol. 2004a;18(4):507–32.

[42] Bordia P, Hunt E, Paulsen N, Tourish D, DiFonzo N. Uncertainty during organizational change: is it all about control? Eur J Work Organizational Psychol. 2004;13(3):345–65.

[43] Bernal D, Campos-Serna J, Tobias A, Vargas-Prada S, Benavides FG, Serra C. Work-related psychosocial risk factors and musculoskeletal disorders in hospital nurses and nursing aides: A systematic review and meta-analysis. Int J Nurs Stud. 2015;52(2):635–48. 25480459 10.1016/j.ijnurstu.2014.11.003

[44] Johansson G, Lundberg O, Lundberg I. Return to work and adjustment latitude among employees on long-term sickness absence. J Occup Rehabil. 2006;16(2):185–95. 16710761 10.1007/s10926-006-9020-9

[45] Stochkendahl MJ, Hasle P, Hansen AF et al. Worker participation in the prevention of musculoskeletal risks at work. In: EU-OSHA. Luxembourg: Publication Office of the European Union; 2022. 10.2802/645868.

[46] Lindström K. User’s guide for the QPSNordic: general nordic questionnaire for psychological and social factors at work. Volume 2000:603. Copenhagen: Nordic Council of Ministers; 2000.

[47] Rizzo JHR, Lirtzman S. Role conflict and ambiguity in complex organizations. Adm Sci Q. 1970;15(2):150–63.

[48] Lang J, Thomas JL, Bliese PD, Adler AB. Job demands and job performance: the mediating effect of psychological and physical strain and the moderating effect of role clarity. J Occup Health Psychol. 2007;12(2):116–24. 17469994 10.1037/1076-8998.12.2.116

[49] Michie S, Williams S. Reducing work related psychological ill health and sickness absence: a systematic literature review. Occup Environ Med. 2003;60(1):3–9. 10.1136/oem.60.1.3 PMC1740370 12499449

[50] Borritz M, Christensen KB, Bültmann U, Rugulies R, Lund T, Andersen I, Villadsen E, Diderichsen F, Kristensen TS. Impact of burnout and psychosocial work characteristics on future Long-Term sickness absence. Prospective results of the Danish PUMA study among human service workers. J Occup Environ Med. 2010;52(10):964–70. 20881631 10.1097/JOM.0b013e3181f12f95

[51] Sundstrup E, Hansen Å, Mortensen E, Clausen T, Rugulies R, Møller A, Andersen L. Retrospectively assessed psychosocial working conditions as predictors of prospectively assessed sickness absence and disability pension among older workers. BMC Public Health. 2018;18(1):1–11. 10.1186/s12889-018-5047-z PMC5773165 29343243

[52] Schmidt S, Roesler U, Kusserow T, Rau R. Uncertainty in the workplace: examining role ambiguity and role conflict, and their link to depression-a meta-analysis. Eur J Work Organizational Psychol. 2014;23(1):91–106.

[53] Borritz BM, Bültmann SU, Rugulies SR, Christensen SK, Villadsen SE, Kristensen ST. Psychosocial work characteristics as predictors for burnout: findings from 3-Year follow up of the PUMA study. J Occup Environ Med. 2005;47(10):1015–25. 16217242 10.1097/01.jom.0000175155.50789.98

[54] Frögéli E, Rudman A, Gustavsson P. The relationship between task mastery, role clarity, social acceptance, and stress: an intensive longitudinal study with a sample of newly registered nurses. Int J Nurs Stud. 2019;91:60. 30677589 10.1016/j.ijnurstu.2018.10.007

[55] Verlinden S, Wynen J, Kleizen B, Verhoest K. Blurred lines: exploring the impact of change complexity on role clarity in the public sector. Rev Public Personnel Adm. 2023;43(3):479–503.

[56] Pollard TM. Changes in mental well-being, blood pressure and total cholesterol levels during workplace reorganization: the impact of uncertainty. Work Stress. 2001;15(1):14–28.

[57] Meyer JP, Allen NJ. A three-component conceptualization of organizational commitment. Hum Resource Manage Rev. 1991;1(1):61–89.

[58] Herscovitch L, Meyer JP. Commitment to organizational change: extension of a Three-Component model. J Appl Psychol. 2002;87(3):474–87. 12090605 10.1037/0021-9010.87.3.474

[59] Begley TM, Czajka JM. Panel analysis of the moderating effects of commitment on job satisfaction, intent to quit, and health following organizational change. J Appl Psychol. 1993;78(4):552–6. 8407703 10.1037/0021-9010.78.4.552

[60] Mowday RT, Porter LW, Steers RM. Employee-organization linkages: the psychology of commitment, absenteeism, and turnover. In. New York, New York;,London, England: Academic; 1982.

[61] Van Der Klink JJL, Blonk RWB, Schene AH, Van Dijk FJH. Reducing long term sickness absence by an activating intervention in adjustment disorders: A cluster randomised controlled design. Occup Environ Med. 2003;60(6):429–37. 12771395 10.1136/oem.60.6.429 PMC1740545

[62] Meyer JP, Stanley DJ, Herscovitch L, Topolnytsky L. Affective, continuance, and normative commitment to the organization: A Meta-analysis of antecedents, correlates, and consequences. J Vocat Behav. 2002;61(1):20–52.

[63] Hausknecht J, Hiller N, Vance R. Work-unit absenteeism: effects of satisfaction, commitment, labor market conditions, and time. Acad Manag J. 2008;51(6):1223–45.

[64] Clausen T, Christensen KB, Borg V. Positive work-related States and long-term sickness absence: A study of register-based outcomes. Scand J Public Health. 2010;38(3suppl):51–8. 21172771 10.1177/1403494809352105

[65] Schalk R. The influence of organizational commitment and health on sickness absenteeism: a longitudinal study. J Nurs Adm Manag. 2011;19(5):596–600. 10.1111/j.1365-2834.2010.01170.x 21749533

[66] Meyer JP, Allen NJ, Topolnytsky L. Commitment in a changing world of work. Can Psychol. 1998;39(1–2):83–93.

[67] Knudsen HK, Aaron Johnson J, Martin JK, Roman PM. Downsizing survival: the experience of work and organizational commitment. Sociol Inq. 2003;73(2):265–83.

[68] Quinlan M, Bohle P. Overstretched and unreciprocated commitment: reviewing research on the occupational health and safety effects of downsizing and job insecurity. Int J Health Serv. 2009;39(1):1–44. 19326777 10.2190/HS.39.1.a

[69] Jain AK, Giga SI, Cooper CL. Stress, health and well-being: the mediating role of employee and organizational commitment. Int J Environ Res Public Health. 2013;10(10):4907–24. 24157512 10.3390/ijerph10104907 PMC3823345

[70] Kelloway EK. Using LISREL for structural equation modeling: a researcher’s guide. Thousand Oaks, Calif: Sage; 1998.

[71] LeBreton JM, Senter JL. Answers to 20 questions about interrater reliability and interrater agreement. Organizational Res Methods. 2008;11(4):815–52.

[72] Hayes AF. Introduction to mediation, moderation, and conditional process analysis: A regression-based approach. Guilford Press; 2013.

[73] Mood C. Logistic regression: why we cannot do what we think we can do, and what we can do about it. Eur Sociol Rev. 2009;26(1):67–82.

[74] Väänänen A, Kalimo R, Toppinen-Tanner S, Mutanen P, Peiró JM, Kivimäki M, Vahtera J. Role clarity, fairness, and organizational climate as predictors of sickness absence: A prospective study in the private sector. Scand J Public Health. 2004;32(6):426–34. 15762027 10.1080/14034940410028136

[75] Datta DK, Guthrie JP, Basuil D, Pandey A. Causes and effects of employee downsizing: A review and synthesis. J Manag. 2010;36:281–348.

[76] Van Der Hulst M. Long workhours and health. Scand J Work Environ Health. 2003;29(3):171–88. 12828387 10.5271/sjweh.720

[77] Marmot M, Feeney A, Shipley M, North F, Syme SL. Sickness absence as a measure of health status and functioning: from the UK Whitehall II study. In. BMJ Publishing Group Ltd; 1995. p. 124. 10.1136/jech.49.2.124 PMC1060095 7798038

[78] Zapf D, Dormann C, Frese M, Quick JC. Longitudinal studies in organizational stress research: A review of the literature with reference to methodological issues. J Occup Health Psychol. 1996;1(2):145–69. 9547043 10.1037//1076-8998.1.2.145

[79] Slany C, Schütte S, Chastang J-F, Parent-Thirion A, Vermeylen G, Niedhammer I. Psychosocial work factors and long sickness absence in Europe. Int J Occup Environ Health. 2014;20(1):16–25. 24176393 10.1179/2049396713Y.0000000048 PMC4137803

[80] Finne LB, Christensen JO, Knardahl S. Psychological and social work factors as predictors of mental distress: A prospective study. 2014. 10.1371/journal.pone.0102514 PMC4105444 25048033

[81] Grønstad A, Kjekshus LE, Tjerbo T et al. Work-related moderators of the relationship between organizational change and sickness absence: a longitudinal multilevel study. BMC Public Health. 2020;20:1–14. 10.1186/s12889-020-09325-w PMC7414577 32770987

[82] Podsakoff PM, Mackenzie SB, Lee J-Y, Podsakoff NP. Common method biases in behavioral research: A critical review of the literature and recommended remedies. J Appl Psychol. 2003;88(5):879–903. 14516251 10.1037/0021-9010.88.5.879

[83] Diez-Roux AV. Bringing context back into epidemiology: variables and fallacies in multilevel analysis. Am J Public Health. 1998;88(2):216–22. 9491010 10.2105/ajph.88.2.216 PMC1508189

[84] Väänänen A, Tordera N, Kivimäki M, Kouvonen A, Pentti J, Linna A, Vahtera J. The role of work group in individual sickness absence behavior. J Health Soc Behav. 2008;49(4):452–67. 19181049 10.1177/002214650804900406

[85] Harter JK, Schmidt FL, Hayes TL. Business-Unit-Level relationship between employee satisfaction, employee engagement, and business outcomes: A Meta-Analysis. J Appl Psychol. 2002;87(2):268–79. 12002955 10.1037/0021-9010.87.2.268

[86] SSB. (2019a). Arbeidsmarkedet for helsepersonnel fram mot 2035 (Labour market prospects for healthcare workers toward 2035). Retrieved from https://www.ssb.no/arbeid-oglonn/artikler-og-publikasjoner/arbeidsmarkedet-for-helsepersonell-fram-mot-2035.

[87] Sainani KL. The problem of multiple testing. PM&R. 2009;1(12):1098–103. 20006317 10.1016/j.pmrj.2009.10.004

