# Supplementary material for: Why downsizing may increase sickness absence: longitudinal fixed effects analyses of the importance of the work environment
Source: BMC Health Serv Res. 2025 Feb 28;25:325. doi: 10.1186/s12913-025-12454-w (PMC11869673; doi:10.1186/s12913-025-12454-w)
Supplement: Supplementary file 1 — Supplementary Material 1. [file 12913_2025_12454_MOESM1_ESM.docx]

| **Supplementary material**  Table A  A random effects multilevel linear analysis of the relationship between unit-level downsizing and unit-level control, role clarity and commitment | | | | | | | | | | |
| --- | --- | --- | --- | --- | --- | --- | --- | --- | --- | --- |
|  |  | 95% CI | |  | 95% CI | |  | 95% CI | |  |
| A work environment for: | Control | LL UL | | Role Clarity | LL UL | | Commitment | LL UL | |  |
| Downsizing next quarter | -0.036 | -0.092 | 0.021 | 0.019 | -0.022 | 0.060 | 0.038 | -0.029 | 0.106 |  |
| Downsizing this quarter | -0.102* | -0.190 | -0.015 | -0.036 | -0.098 | 0.026 | -0.196*** | -0.298 | -0.093 |  |
| Downsizing previous quarter | -0.280*** | -0.435 | -0.124 | 0.031 | -0.079 | 0.141 | -0.209* | -0.391 | -0.027 |  |
| No. of employees | -0.005*** | -0.007 | -0.003 | 0.000 | -0.001 | 0.001 | -0.000 | -0.002 | 0.001 |  |
| Female | 0.132* | 0.005 | 0.260 | 0.083* | 0.011 | 0.154 | 0.021 | -0.098 | 0.140 |  |
| Salary | 0.000*** | 0.000 | 0.001 | -0.000 | -0.000 | 0.000 | 0.001*** | 0.001 | 0.001 |  |
| Age | 0.002 | -0.004 | 0.007 | 0.004** | 0.001 | 0.008 | -0.003 | -0.008 | 0.003 |  |
| Multiple job holder | -0.170* | -0.306 | -0.035 | -0.064 | -0.156 | 0.028 | -0.336*** | -0.488 | -0.184 |  |
| Temporary contracts | 0.066 | -0.089 | 0.221 | -0.029 | -0.127 | 0.070 | 0.191* | 0.028 | 0.354 |  |
| Physician | -0.372*** | -0.535 | -0.209 | -0.131** | -0.221 | -0.041 | -0.676*** | -0.825 | -0.527 |  |
| Other patient-related position | 0.096 | -0.030 | 0.222 | -0.044 | -0.109 | 0.021 | -0.147** | -0.255 | -0.040 |  |
| Administration/management | 0.360*** | 0.229 | 0.492 | -0.134*** | -0.203 | -0.065 | -0.240*** | -0.354 | -0.125 |  |
| Kitchen/cleaning/orderly | 0.546*** | 0.360 | 0.731 | 0.040 | -0.057 | 0.136 | 0.137 | -0.024 | 0.297 |  |
| Other operations | 0.542*** | 0.346 | 0.738 | -0.072 | -0.181 | 0.038 | -0.125 | -0.306 | 0.057 |  |
| Other | 1.286*** | 1.067 | 1.504 | -0.055 | -0.175 | 0.066 | -0.065 | -0.264 | 0.135 |  |
| _cons | 2.540*** | 2.234 | 2.845 | 4.275*** | 4.098 | 4.452 | 3.761*** | 3.468 | 4.054 |  |
| Random-effects Parameters |  |  | |  |  | |  |  | |  |
|  | Variance | Sdt. Err. | | Variance | Sdt. Err. | | Variance | Sdt. Err. | |  |
| Level 2: work-unit | 0.125 | 0.008 | | 0.230 | 0.012 | | 0.045 | 0.003 | |  |
| Level 1: work-unit-quater | 0.110 | 0.003 | | 0.069 | 0.002 | | 0.040 | 0.001 | |  |
| *Note.* N work-units: 876. N observations 2 500. | | | | |  | |  |  | |  |

| Table B  *A random effects linear probability model of the relationship between unit-level downsizing and short-term sickness absence without and with control for work environment factors* | | | | | | | | | | | | | | | |
| --- | --- | --- | --- | --- | --- | --- | --- | --- | --- | --- | --- | --- | --- | --- | --- |
|  |  | 95% CI | |  | 95% CI | |  |  | |  | 95% CI | |  | 95% CI | |
| A work environment for: | Model 0 | LL | UL | Model 1 | LL | UL | Model 2 | LL | UL | Model 3 | LL | UL | Model 4 | LL | UL |
| Downsizing next quarter | -0.041*** | -0.059 | -0.023 | -0.041*** | -0.059 | -0.023 | -0.041*** | -0.059 | -0.023 | -0.041*** | -0.059 | -0.023 | -0.041*** | -0.059 | -0.023 |
| Downsizing this quarter | 0.036*** | 0.017 | 0.055 | 0.036*** | 0.017 | 0.054 | 0.035*** | 0.016 | 0.054 | 0.036*** | 0.017 | 0.055 | 0.035*** | 0.017 | 0.054 |
| Downsizing previous  quarter | 0.046*** | 0.019 | 0.073 | 0.043** | 0.016 | 0.071 | 0.045** | 0.018 | 0.072 | 0.046*** | 0.019 | 0.073 | 0.043** | 0.016 | 0.070 |
| Female | 0.033*** | 0.022 | 0.044 | 0.034*** | 0.022 | 0.045 | 0.034*** | 0.022 | 0.045 | 0.033*** | 0.022 | 0.044 | 0.034*** | 0.022 | 0.045 |
| Salary | -0.000*** | -0.000 | -0.000 | -0.000*** | -0.000 | -0.000 | -0.000*** | -0.000 | -0.000 | -0.000*** | -0.000 | -0.000 | -0.000*** | -0.000 | -0.000 |
| Age | -0.002*** | -0.003 | -0.002 | -0.002*** | -0.003 | -0.002 | -0.002*** | -0.003 | -0.002 | -0.002*** | -0.003 | -0.002 | -0.002*** | -0.003 | -0.002 |
| Multiple job holder | 0.031*** | 0.017 | 0.046 | 0.030*** | 0.016 | 0.044 | 0.031*** | 0.017 | 0.045 | 0.031*** | 0.017 | 0.046 | 0.030*** | 0.015 | 0.044 |
| Temporary contracts | -0.012 | -0.023 | 0.000 | -0.012 | -0.023 | 0.000 | -0.011 | -0.023 | 0.000 | -0.012 | -0.023 | 0.000 | -0.011 | -0.023 | 0.000 |
| Physician | -0.172*** | -0.195 | -0.148 | -0.178*** | -0.201 | -0.155 | -0.175*** | -0.198 | -0.151 | -0.171*** | -0.195 | -0.148 | -0.178*** | -0.201 | -0.155 |
| Other patient-related position | -0.016* | -0.032 | -0.000 | -0.018* | -0.033 | -0.002 | -0.014 | -0.030 | 0.001 | -0.016* | -0.032 | -0.000 | -0.016* | -0.032 | -0.001 |
| Administration/  management | -0.084*** | -0.101 | -0.066 | -0.086*** | -0.103 | -0.069 | -0.080*** | -0.097 | -0.063 | -0.084*** | -0.101 | -0.066 | -0.083*** | -0.100 | -0.065 |
| Kitchen/cleaning/  orderly | 0.118*** | 0.083 | 0.152 | 0.119*** | 0.085 | 0.153 | 0.125*** | 0.091 | 0.160 | 0.118*** | 0.083 | 0.152 | 0.123*** | 0.089 | 0.157 |
| Other operations | -0.071*** | -0.099 | -0.044 | -0.073*** | -0.101 | -0.046 | -0.065*** | -0.092 | -0.038 | -0.071*** | -0.099 | -0.044 | -0.068*** | -0.095 | -0.041 |
| Other | -0.244*** | -0.275 | -0.213 | -0.245*** | -0.276 | -0.215 | -0.233*** | -0.264 | -0.201 | -0.244*** | -0.275 | -0.213 | -0.236*** | -0.267 | -0.204 |
| Commitment |  |  |  | -0.024*** | -0.034 | -0.014 |  |  |  |  |  |  | -0.029*** | -0.041 | -0.017 |
| Control |  |  |  |  |  |  | -0.018*** | -0.029 | -0.008 |  |  |  | -0.013* | -0.023 | -0.002 |
| Role Clarity |  |  |  |  |  |  |  |  |  | 0.001 | -0.017 | 0.018 | 0.029** | 0.009 | 0.049 |
| _cons | 0.584*** | 0.558 | 0.610 | 0.677*** | 0.631 | 0.723 | 0.635*** | 0.597 | 0.674 | 0.580*** | 0.498 | 0.662 | 0.602*** | 0.519 | 0.686 |
| Random-effects Parameters | |  | |  |  | |  |  | |  |  | |  |  | |
|  | Variance | Sdt. Err. | | Variance | Sdt. Err. | | Variance | Sdt. Err. | | Variance | Sdt. Err. | | Variance | Sdt. Err. | |
| Level 3: work-unit | 0.008 | 0.001 | | 0.008 | 0.001 | | 0.008 | 0.001 | | 0.008 | 0.001 | | 0.007 | 0.001 | |
| Level 2: employee | 0.041 | 0.001 | | 0.041 | 0.001 | | 0.041 | 0.001 | | 0.041 | 0.001 | | 0.041 | 0.001 | |
| Level 1: employee-quater | 0.180 | 0.001 | | 0.181 | 0.001 | | 0.181 | 0.001 | | 0.181 | 0.001 | | 0.181 | 0.001 | |
| *Note.* N work-units: 900. N employees 19 173. N observations 83 570.  * p<0.05, ** p<0.01, *** p<0.001 | | | | | | | | | |  |  | |  |  | |

| Table C  *A random effects linear probability model of the relationship between unit-level downsizing and long-term sickness absence without and with control for work environment factors* | | | | | | | | | | | | | | | |
| --- | --- | --- | --- | --- | --- | --- | --- | --- | --- | --- | --- | --- | --- | --- | --- |
|  |  | 95% CI | |  | 95% CI | |  |  | |  | 95% CI | |  | 95% CI | |
| A work environment for: | Model 0 | LL UL | | Model 1 | LL UL | | Model 2 | LL UL | | Model 3 | LL UL | | Model 4 | LL UL | |
|  |  |  |  |  |  |  |  |  |  |  |  |  |  |  |  |
| Downsizing next quarter | -0.000 | -0.012 | 0.012 | -0.000 | -0.012 | 0.012 | -0.000 | -0.012 | 0.011 | -0.000 | -0.012 | 0.012 | -0.000 | -0.012 | 0.012 |
| Downsizing this quarter | -0.002 | -0.014 | 0.011 | -0.002 | -0.014 | 0.011 | -0.002 | -0.015 | 0.010 | -0.002 | -0.014 | 0.011 | -0.002 | -0.015 | 0.010 |
| Downsizing previous  quarter | 0.023* | 0.004 | 0.041 | 0.021* | 0.002 | 0.039 | 0.022* | 0.003 | 0.040 | 0.022* | 0.004 | 0.041 | 0.021* | 0.002 | 0.039 |
| Female | 0.047*** | 0.041 | 0.054 | 0.048*** | 0.041 | 0.054 | 0.048*** | 0.041 | 0.054 | 0.047*** | 0.041 | 0.054 | 0.048*** | 0.041 | 0.054 |
| Salary | -0.000*** | -0.000 | -0.000 | -0.000*** | -0.000 | -0.000 | -0.000*** | -0.000 | -0.000 | -0.000*** | -0.000 | -0.000 | -0.000*** | -0.000 | -0.000 |
| Age | 0.000* | 0.000 | 0.001 | 0.000* | 0.000 | 0.001 | 0.000* | 0.000 | 0.001 | 0.000* | 0.000 | 0.001 | 0.000* | 0.000 | 0.001 |
| Multiple job holder | 0.007 | -0.002 | 0.016 | 0.006 | -0.003 | 0.015 | 0.006 | -0.003 | 0.015 | 0.007 | -0.002 | 0.016 | 0.006 | -0.003 | 0.015 |
| Temporary contracts | -0.031*** | -0.039 | -0.024 | -0.031*** | -0.038 | -0.023 | -0.031*** | -0.038 | -0.023 | -0.031*** | -0.039 | -0.024 | -0.030*** | -0.038 | -0.023 |
| Physician | -0.024*** | -0.036 | -0.012 | -0.029*** | -0.041 | -0.016 | -0.027*** | -0.039 | -0.015 | -0.025*** | -0.037 | -0.013 | -0.029*** | -0.042 | -0.017 |
| Other patient-related position | 0.006 | -0.003 | 0.014 | 0.004 | -0.004 | 0.012 | 0.007 | -0.002 | 0.015 | 0.006 | -0.003 | 0.014 | 0.005 | -0.003 | 0.013 |
| Administration/  management | -0.009 | -0.018 | 0.000 | -0.011* | -0.020 | -0.002 | -0.006 | -0.015 | 0.004 | -0.009 | -0.018 | 0.000 | -0.008 | -0.017 | 0.002 |
| Kitchen/cleaning/orderly | 0.110*** | 0.093 | 0.126 | 0.110*** | 0.094 | 0.126 | 0.116*** | 0.099 | 0.132 | 0.110*** | 0.094 | 0.126 | 0.114*** | 0.098 | 0.131 |
| Other operations | 0.006 | -0.009 | 0.021 | 0.005 | -0.010 | 0.020 | 0.012 | -0.003 | 0.027 | 0.006 | -0.009 | 0.021 | 0.010 | -0.005 | 0.025 |
| Other | -0.036*** | -0.053 | -0.019 | -0.036*** | -0.053 | -0.020 | -0.025** | -0.042 | -0.007 | -0.036*** | -0.053 | -0.020 | -0.027** | -0.044 | -0.009 |
| Commitment |  |  |  | -0.013*** | -0.019 | -0.007 |  |  |  |  |  |  | -0.013*** | -0.020 | -0.006 |
| Control |  |  |  |  |  |  | -0.013*** | -0.018 | -0.007 |  |  |  | -0.010*** | -0.015 | -0.004 |
| Role Clarity |  |  |  |  |  |  |  |  | 0 | -0.005 | -0.016 | 0.005 | 0.008 | -0.004 | 0.020 |
| _cons | 0.100*** | 0.085 | 0.115 | 0.151*** | 0.124 | 0.179 | 0.133*** | 0.113 | 0.154 | 0.124*** | 0.075 | 0.173 | 0.141*** | 0.091 | 0.191 |
| Random-effects Parameters | |  | |  |  | |  |  | |  |  | |  |  | |
|  | Variance | Sdt. Err. | | Variance | Sdt. Err. | | Variance | Sdt. Err. | | Variance | Sdt. Err. | | Variance | Sdt. Err. | |
| Level 3: work-unit | 0.001 | 0.000 | | 0.001 | 0.000 | | 0.001 | 0.000 | | 0.001 | 0.000 | | 0.001 | 0.000 | |
| Level 2: employee | 0.010 | 0.000 | | 0.010 | 0.000 | | 0.010 | 0.000 | | 0.010 | 0.000 | | 0.010 | 0.000 | |
| Level 1: employee-quater | 0.089 | 0.000 | | 0.089 | 0.000 | | 0.089 | 0.000 | | 0.089 | 0.000 | | 0.089 | 0.000 | |
| *Note.* N work-units: 900. N employees 19 173. N observations 83 570.  * p<0.05, ** p<0.01, *** p<0.001 | | | | | | |  |  | |  |  | |  |  | |
|  | |  | |  |  | |  |  | |  |  | |  |  | |
|  |  |  | |  |  | |  |  | |  |  | |  |  | |
