# Supplementary material for: Why downsizing may increase sickness absence: longitudinal fixed effects analyses of the importance of the work environment
Source: BMC Health Serv Res. 2025 Feb 28;25:325. doi: 10.1186/s12913-025-12454-w (PMC11869673; doi:10.1186/s12913-025-12454-w)
Supplement: Supplementary file 2 — Supplementary Material 2. [file 12913_2025_12454_MOESM2_ESM.docx]

| **Table A: A random effects structural equation model** | |  |  |  |  |  |
| --- | --- | --- | --- | --- | --- | --- |
| **Downsizing -- > Commitment -- > sickness absence** | |  |  |  |  |  |
|  | **short-term sickness absence** | | | **long-term sickness absence** | | |
|  | coef. | 95% CI |  | coef. | 95% CI |  |
| **Predicting short-term sickness absence^1^** |  |  |  |  |  |  |
| Downsizing next quarter | 0.05 | -0.15 | 0.00 | 0.07 | 0.13 | 0.00 |
| Downsizing this quarter | 0.21 | 0.10 | 0.32 | -0.02 | -0.17 | 0.13 |
| Downsizing previous quarter | 0.24 | 0.09 | 0.39 | 0.20 | 0.01 | 0.40 |
| Commitment | -0.13 | -0.18 | -0.07 | -0.14 | -0.21 | -0.08 |
| **Predicting comittment^2^** |  |  |  |  |  |  |
| Downsizing next quarter | -0.02 | -0.11 | 0.06 | -0.02 | -0.11 | 0.06 |
| Downsizing this quarter | -0.17 | -0.26 | -0.08 | -0.17 | -0.26 | -0.08 |
| Downsizing previous quarter | -0.29 | -0.44 | -0.15 | -0.29 | -0.44 | -0.15 |
| **Estimated indirect effect via comittment** |  |  |  |  |  |  |
| Downsizing next quarter | 0.00 | -0.01 | 0.01 | 0.00 | -0.01 | 0.02 |
| Downsizing this quarter | 0.02 | 0.01 | 0.04 | 0.02 | 0.01 | 0.04 |
| Downsizing previous quarter | 0.04 | 0.01 | 0.06 | 0.04 | 0.01 | 0.07 |
| **Downsizing -- > control -- > sickness absence** |  |  |  |  |  |  |
|  | **Short-term sickness absence** | | | **Long-term sickness absence** | | |
|  | coef. | 95% CI |  | coef. | 95% CI |  |
| **Predicting short-term sickness absence^1^** |  |  |  |  |  |  |
| Downsizing next quarter | -0.25 | -0.36 | -0.15 | -0.01 | -0.16 | 0.14 |
| Downsizing this quarter | 0.21 | 0.10 | 0.31 | -0.02 | -0.17 | 0.13 |
| Downsizing previous quarter | 0.25 | 0.10 | 0.40 | 0.21 | 0.02 | 0.41 |
| Control | -0.09 | -0.15 | -0.04 | -0.15 | -0.21 | -0.08 |
| **Predicting control^2^** |  |  |  |  |  |  |
| Downsizing next quarter | -0.09 | -0.11 | -0.07 | -0.10 | -0.21 | 0.00 |
| Downsizing this quarter | -0.15 | -0.17 | -0.12 | -0.08 | -0.20 | 0.05 |
| Downsizing previous quarter | -0.12 | -0.15 | -0.08 | -0.25 | -0.44 | -0.07 |
| **Estimated indirect effect via control** |  |  |  |  |  |  |
| Downsizing next quarter | 0.01 | 0.00 | 0.02 | 0.02 | 0.00 | 0.03 |
| Downsizing this quarter | 0.01 | -0.01 | 0.02 | 0.01 | -0.01 | 0.03 |
| Downsizing previous quarter | 0.02 | 0.00 | 0.05 | 0.04 | 0.01 | 0.07 |
| **Downsizing -- > role clarity -- > sickness absence** | |  |  |  |  |  |
|  | **Short-term sickness absence** | | | **Long-term sickness absence** | | |
|  | coef. | 95% CI |  | coef. | 95% CI |  |
| **Predicting short-term sickness absence^1^** | 0.00 | 0.00 | 0.00 | 0.00 | 0.00 | 0.00 |
| Downsizing next quarter | -0.25 | -0.36 | -0.14 | -0.01 | -0.16 | 0.14 |
| Downsizing this quarter | 0.21 | 0.10 | 0.32 | -0.02 | -0.17 | 0.13 |
| Downsizing previous quarter | 0.25 | 0.10 | 0.40 | 0.22 | 0.03 | 0.41 |
| Role clarity | 0.01 | -0.09 | 0.11 | -0.06 | -0.18 | 0.06 |
| **Predicting role clarity^2^** | 0.00 | 0.00 | 0.00 | 0.00 | 0.00 | 0.00 |
| Downsizing next quarter | -0.06 | -0.11 | -0.01 | -0.06 | -0.11 | -0.01 |
| Downsizing this quarter | -0.08 | -0.14 | -0.03 | -0.08 | -0.14 | -0.03 |
| Downsizing previous quarter | -0.10 | -0.18 | -0.02 | -0.10 | -0.18 | -0.02 |
| **Estimated indirect effect via role clarity** | 0.00 | 0.00 | 0.00 | 0.00 | 0.00 | 0.00 |
| Downsizing next quarter | 0.00 | -0.01 | 0.01 | 0.00 | 0.00 | 0.01 |
| Downsizing this quarter | 0.00 | -0.01 | 0.01 | 0.01 | -0.01 | 0.02 |
| Downsizing previous quarter | 0.00 | -0.01 | 0.01 | 0.01 | -0.01 | 0.02 |
| ^1^ Analyses is nested within employee and work unit and controlled for gender, sallary, age, multiple contracts, temporary contract, and postition | | | | | | |
| ^1^ N= 83 570 observations (employee-quater) |  |  |  |  |  |  |
| ^2^ N= 2 692 observations (unit-quater) |  |  |  |  |  |  |
